# Tannins as Therapeutic Agents for Ulcerative Colitis: Mechanisms and Prospects in Regulating Gut Inflammatory-Oxidative Homeostasis

**DOI:** 10.3390/molecules31071116

**Published:** 2026-03-28

**Authors:** Yanling Li, Can Sun, Fuqi Hao, Yichi Wang, Jianxi Zhu, Yujiao Ming, Miaomiao Tian, Le Li, Huiqin Qian

**Affiliations:** 1 College of Pharmacy, North Henan Medical University, Xinxiang 453003, China; 2 College of Pharmacy, Henan Medical University, Xinxiang 453003, China

**Keywords:** tannins, ulcerative colitis, inflammation, oxidative stress, gut microbiota

## Abstract

Ulcerative colitis (UC) is a multifactorial disease characterized by chronic intestinal inflammation and disrupted oxidative balance, significantly impairing patients’ quality of life. Tannins, a class of polyphenolic compounds widely distributed in plants, have demonstrated notable therapeutic potential against UC due to their inherent antioxidant and anti-inflammatory properties. This study employs a systematic literature review of databases, including PubMed and Web of Science, to investigate the molecular mechanisms by which tannins restore intestinal inflammatory and oxidative homeostasis. The findings indicate that tannins directly scavenge reactive oxygen species (ROS) via their polyphenolic structure, mitigate oxidative damage, upregulate antioxidant enzyme expression, suppress pro-inflammatory cytokine secretion, and preserve intestinal barrier integrity. Despite their significant therapeutic promise, challenges such as low bioavailability and structural complexity remain. Future research should prioritize bioavailability enhancement, clarification of structure-activity relationships, and translational studies to facilitate the clinical application of tannin-based therapies for UC.

## 1. Introduction

**Figure 1:**
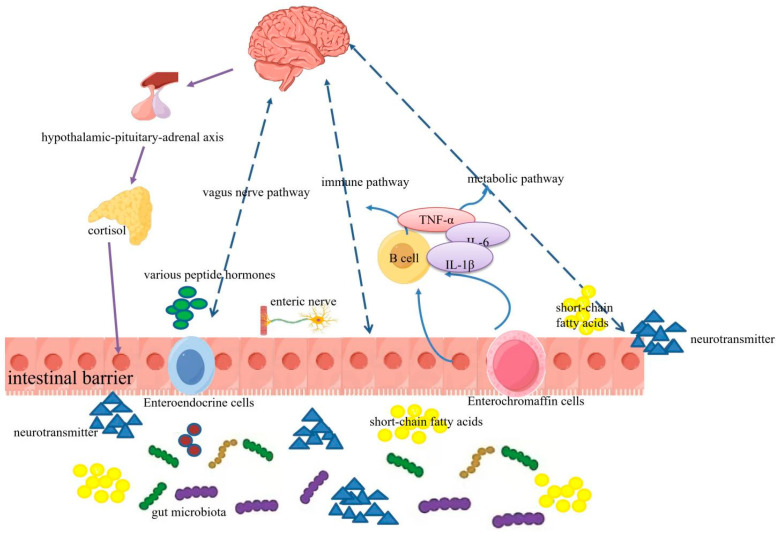
Schematic representation of IBD pathogenesis. This figure shows the bidirectional regulatory network between the brain, intestine and gut microbiota through neural, endocrine, immune and metabolic pathways; the core regulatory pathways are marked with blue dashed lines, and cells and molecules with different functions are identified by differentiated graphics. Different colors represent distinct components of gut microbiota and signaling molecules: red circular, green rod-shaped, purple rod-shaped, and brown curved rod-shaped symbols represent different types of gut microbiota; blue triangular symbols represent neurotransmitters; yellow clustered symbols represent short-chain fatty acids.

Inflammatory bowel diseases (IBD) are a complex group of conditions characterized by chronic intestinal inflammation. Their pathogenesis involves genetic factors, dysbiosis of the gut microbiota, immune responses, and environmental influences [1,2,3], the intricate regulatory networks and key molecular mediators of which are illustrated in Figure 1. UC is a complex inflammatory bowel disease characterized by a chronic, relapsing-remitting course. This persistent state of inflammation not only underpins the primary intestinal pathology but also elevates the long-term risk of complications such as colorectal cancer [4,5]. Clinically, UC manifests with a spectrum of debilitating symptoms, most notably abdominal pain, hemorrhagic diarrhea, and the passage of bloody stools [6]. These clinical features are the direct consequence of profound disruptions at the molecular and cellular level, including intense oxidative stress, an accumulation of pro-inflammatory mediators, and a compromised intestinal barrier integrity [7,8]. Thus, the clinical picture of UC is a manifestation of these underlying pathophysiological disturbances. Current clinical management faces significant therapeutic challenges: although aminosalicylates (e.g., mesalamine), glucocorticoids, and antibiotics [9,10] provide symptomatic relief, their efficacy remains suboptimal with frequent adverse effects [11]. Consequently, developing novel targeted therapeutics constitutes a major focus in translational gastroenterology.

**Figure 2:**
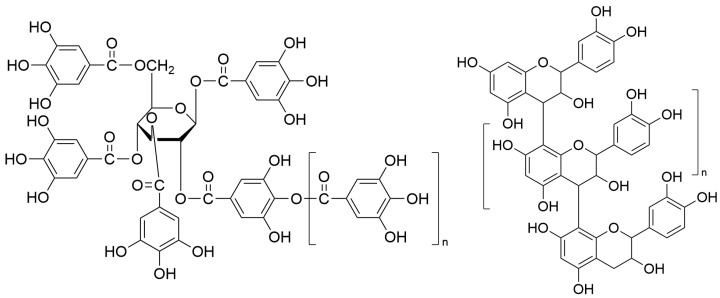
The molecular structure of hydrolysable tannin and condensed tannin. The left figure shows the molecular structure of hydrolysable tannin, the right figure shows the molecular structure of condensed tannin.

Tannins, also referred to as plant polyphenols, are widely distributed throughout the vegetal species kingdom [12,13]. These metabolites are particularly abundant in pomegranates [14,15], grapes [16], and persimmons [17,18]. Persimmon tannins are considered ideal models for studying the bioactivities of tannins. Variations in structural complexity and polymerization degree among botanical sources critically influence their therapeutic efficacy against UC. Chemically, tannins are categorized into two principal classes: hydrolysable tannins [19], which are Gallic/ellagic acid derivatives esterified to glucose/polyol cores, undergoing cleavage under acidic/alkaline/ enzymatic conditions to exert antimicrobial, antidiarrheal (via astringency), and potent antioxidant effects, and condensed tannins, which are Flavan-3-ol oligomers polymerized via C4→C8/C6 linkages (proanthocyanidins), irreversibly condensing into insoluble phlobaphenes under strong acidic environments [20,21]. In Figure 2, these two classes are distinguished by fundamental differences in their molecular architecture, which in turn govern their distinct physicochemical properties and biological activities.

Tannins have garnered significant attention for their potent bioactivities in ameliorating UC, functioning through multi-target mechanisms: modulation of pro-inflammatory cytokines, suppression of inflammatory signaling cascades, enhancement of antioxidant defenses, restoration of gut microbial equilibrium, and reinforcement of intestinal barrier integrity [22,23]. Their therapeutic mechanisms include antioxidant duality [24], acting as electron donors to quench free radicals while depleting ambient oxygen via redox cycling. Antimicrobial potency [25,26]: Coagulating microbial protoplasm and inhibiting essential enzymes, effectively suppressing pathogens like *Vibrio cholerae* and *Escherichia coli*. Antitumor potential [27,28]: Enhancing DNA damage repair, activating NK cell cytotoxicity, and inhibiting PI3K/Akt oncogenic pathways. Given their favorable efficacy-to-safety profile, tannins represent emerging candidates for UC pharmacotherapy [29]. Due to its good therapeutic effects and low side effects, tannin has become a hot topic in the research and development of new drugs for UC [30,31]. In the future, in-depth research into the mechanism of action of tannin and its potential application in the treatment of UC using modern scientific methods will provide an important basis for the development of safer and more effective treatment strategies.

However, despite the burgeoning interest in tannins, existing research has largely concentrated on their isolated pharmacological effects, such as their antioxidant or anti-inflammatory properties per se. Compared with similar studies [32,33,34], this research innovatively integrates the multi-targeted intervention effects of tannins on inflammatory mediators, oxidative stress, and gut microbiota. This mechanism complements studies on postbiotic therapy, enhancing barrier function through immune-metabolic pathways. At the same time, the plant-derived nature of tannins eliminates the risk of microbial contamination. Compared to the therapeutic strategy of Traditional Chinese Medicine formulas that modulate the gut microbiota-immune axis through multiple components [35,36], this study focuses more on the precise regulation of oxidative stress and inflammatory pathways by tannins, offering new insights for precision medicine in UC.

This fragmented approach has resulted in a lack of systematic synthesis regarding their holistic regulation of the core pathological driver of UC: the intricate balance between intestinal inflammation and oxidative stress. Consequently, the overarching mechanisms by which tannins integrate these multiple pathways to restore gut homeostasis remain incompletely understood. Therefore, the primary objective of this review is to provide a comprehensive and systematic overview of the molecular mechanisms through which tannins re-establish the inflammatory-oxidative balance in the gut, thereby offering a foundational framework for future research and therapeutic development in UC.

## 2. Literature Retrieval and Screening Methods

### 2.1. Search Strategy

A comprehensive literature search was conducted across multiple electronic databases, including PubMed, Web of Science, Google Scholar, ScienceDirect, China National Knowledge Infrastructure (CNKI), and Wanfang Data, to identify relevant studies published from January 1961 to December 2025. The search strategy was designed to capture all potentially eligible articles by combining terms related to tannins, ulcerative colitis, and the underlying mechanisms of inflammation and oxidative stress. For instance, the search query used for PubMed was as follows: (“Tannins”[Mesh] OR “Tannin”[Title/Abstract] OR “Tannic Acid”[Title/Abstract] OR “Polyphenol”[Mesh] OR “Polyphenol”[Title/Abstract]) AND (“Colitis, Ulcerative”[Mesh] OR “Ulcerative Colitis”[Title/Abstract] OR “Inflammatory Bowel Diseases”[Mesh] OR “IBD”[Title/Abstract]) AND (“Inflammation”[Mesh] OR “Oxidative Stress”[Mesh] OR “Antioxidants”[Mesh] OR “Cytokines”[Mesh] OR “NF-kappa B”[Mesh]). This search syntax, employing Boolean operators (AND, OR), was adapted for each database to ensure a systematic and reproducible retrieval process.

### 2.2. Study Selection and Inclusion Criteria

Study selection followed a two-phase process, performed independently by two reviewers (Y.L. and C.S.). In the first phase, the titles and abstracts of all retrieved records were screened against pre-defined inclusion criteria. Articles were considered eligible if they: (1) were original research articles (e.g., in vitro, in vivo, or clinical studies) published in English or Chinese, 65 articles; (2) investigated the effects of tannins, polyphenols, or their derivatives (e.g., gallic acid, ellagic acid) on intestinal inflammation or oxidative stress, 220 articles; (3) reported outcomes related to inflammatory mediators, antioxidant enzymes, gut microbiota, or intestinal barrier function, 343 articles. Review articles, conference abstracts, case reports, and editorials were excluded at this stage.

In the second phase, the full texts of potentially relevant articles were retrieved and independently assessed by the same two reviewers for final inclusion. Disagreements between reviewers at either phase were resolved through discussion or, if necessary, by consultation with a third reviewer (H.Q.). The reasons for excluding any full-text articles were documented. This systematic approach ensured a transparent and reproducible selection of literature for this review.

## 3. Mechanisms Underlying the Therapeutic Effects of Tannins in UC

### 3.1. Modulation of Pro-Inflammatory Cytokine

UC pathogenesis is intrinsically linked to dysregulated inflammation, wherein sustained inflammatory responses drive disease progression. Under inflammatory conditions, the body generates excessive pro-inflammatory mediators, including tumor necrosis factor-α (TNF-α), interleukin-1β (IL-1β), and interleukin-6 (IL-6) [37,38]. Concurrently, a self-perpetuating vicious cycle emerges between oxidative stress and inflammation. Critically, aberrant activation of key signaling pathways, particularly NF-*κ*B [39,40] and PI3K/Akt [41,42] inflammatory cascades that exacerbate intestinal mucosal damage.

Tannins exert therapeutic effects through multi-target and multi-level synergistic mechanisms, precisely targeting the inflammatory pathological process of UC: on one hand, they inhibit the activation of immune cells such as macrophages, thereby reducing the synthesis and release of pro-inflammatory factors such as TNF-*α*, IL-1*β*, and IL-6, and block the vicious cycle of oxidative stress and inflammation by scavenging reactive oxygen species (ROS) [43,44,45]; on the other hand, they target and regulate key signaling pathways such as NF-*κ*B and PI3K/Akt, downregulating the transcription of pro-inflammatory genes while upregulating the expression of anti-inflammatory factors like IL-10, thereby achieving bidirectional regulation of the inflammatory cascade reaction [43,46,47]. Basic research and clinical evidence indicate [48,49] that tannins can not only directly intervene in the inflammatory factor network but also reshape the inflammation-oxidation balance by blocking pathogen invasion and regulating immune cell function, providing a natural intervention strategy for UC treatment that is both broad-spectrum and specific.

The regulatory effects of tannins derived from diverse vegetal species on key inflammatory mediators in UC, along with their corresponding action mechanisms and validated experimental models, are systematically summarized in Table 1. These natural tannins exert species-specific anti-inflammatory activities by targeting critical pro-inflammatory factors (e.g., TNF-α, IL-6, IL-1β) and canonical inflammatory signaling pathways, with their biological effects verified in a variety of well-characterized in vitro cell models, in vivo animal models and ex vivo microbial culture systems, which provides a solid experimental basis for further elucidating the anti-inflammatory mechanism of tannins in UC.

#### 3.1.1. Suppression of Pro-Inflammatory Mediator Secretion

UC pathogenesis is intimately implicated in immune dysregulation, with inflammatory disruption serving as the pivotal driver of disease progression. During inflammatory cascades, activated immune cells—particularly macrophages and monocytes—release substantial quantities of pro-inflammatory cytokines, including TNF-α, IL-1β, and IL-6 [50,51]. These mediators perpetuatively amplify inflammation through epithelial barrier disruption, enterocyte apoptosis, and stromal activation [52,53,54,55]. Collectively, such pathological alterations culminate in characteristic UC manifestations: mucosal ulcerations, abdominal pain, diarrhea with mucoid bloody stools, and histopathological crypt destruction [56].

Natural polyphenolic tannins exhibit multi-targeted regulation of inflammatory cascades [57]. Zhen Li [58] demonstrated that tannic acid-loaded nanoparticles, pathogen interception, cytokine suppression, and oxidative homeostasis. Concurrently, Piazza S et al. [57] discovered through numerous in vivo experiments that orally administered hydrolyzed tannins can decrease the levels of cytokines secreted by Th cells, encompassing IFN-γ, IL-17, and IL-4. The mechanism of action encompasses the inhibition of inflammatory transcription factors (NF-*κ*B, NFAT), enzymes (MAPKs, COX-2, iNOS), and ion channels. This validates the potential of the multi-target anti-inflammatory mechanisms of tannins, thereby halting the progression of inflammation, facilitating UC intestinal mucosal repair, alleviating disease symptoms, and furnishing a theoretical foundation for the treatment of UC.

Further support for the anti-inflammatory potential of tannins comes from clinical investigations beyond the immediate context of IBD. For instance, in a randomized placebo-controlled trial by Molino et al. [59], hospitalized COVID-19 patients receiving oral tannin supplements showed no significant improvement in clinical symptoms or gut microbiota α-diversity by day 14. However, the intervention did markedly attenuate systemic inflammation, as measured by specific inflammatory markers. Although COVID-19 and UC have distinct etiologies, they share a common pathological feature of dysregulated and excessive inflammatory responses. Therefore, this finding from a human trial provides valuable, albeit indirect, evidence of tannins’ ability to modulate systemic inflammation in vivo. It supports the hypothesis that the anti-inflammatory mechanisms observed in cell and animal models of UC may be translatable to human inflammatory conditions, reinforcing the rationale for developing tannin-based therapies for UC. Mechanistically, tannins exert dual protective effects; hydrophobic moieties bind microbial surface adhesins, inhibiting enteric pathogen invasion, thereby blocking the invasion of pathogenic bacteria into the intestine, to alleviate cellular inflammatory response [60,61]. Furthermore, Tao H et al. [62] demonstrated that employing immunohistochemistry (IHC) or immunofluorescence (IF) techniques enables targeted detection of specific inflammatory cell markers (e.g., CD68 for macrophages). This approach permits quantitative analysis of phenotypic alterations in distinct inflammatory cell populations before and after tannin intervention, thereby elucidating the mechanistic impact of tannins on the inflammation-oxidation equilibrium.

**Figure 3:**
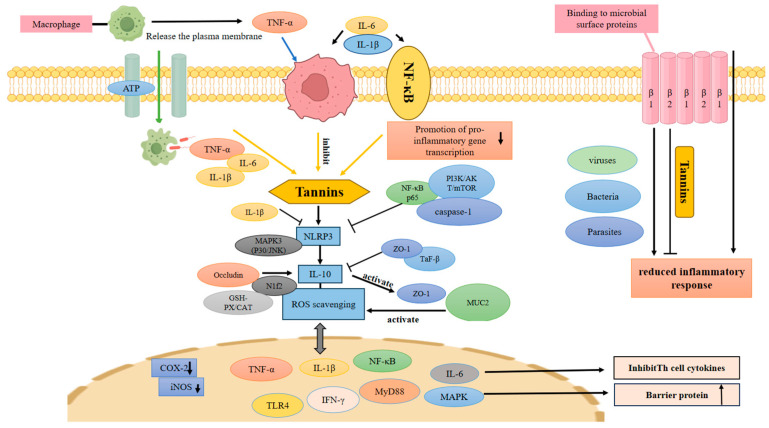
Schematic diagram of the molecular mechanism of tannins in regulating inflammation and immune response. The upper phospholipid bilayer represents the host cell plasma membrane, and the right phospholipid bilayer represents the microbial membrane (bacteria, viruses, parasites). Under pathogenic stimulation, macrophages are activated and release a variety of pro-inflammatory factors, which further trigger intracellular inflammatory signaling pathways, including PI3K/Akt/NF-*κ*B, MAPK, and NLRP3 inflammasome, ultimately leading to severe inflammatory responses. Tannins serve as the key regulatory component to inhibit the activation of macrophages and the production of pro-inflammatory cytokines. Meanwhile, tannins directly act on microbial surface structures to reduce pathogenic stimulation. Through inhibiting multiple inflammatory signaling pathways, upregulating antioxidant enzymes and enhancing the expression of tight junction proteins, tannins effectively alleviate oxidative stress and inflammatory damage, maintain cellular barrier function, and finally exert significant anti-inflammatory and immunomodulatory effects. Arrow explanation: Downward arrows (↓) indicate downregulation or inhibition; upward arrows (↑) indicate upregulation or activation; solid arrows (→) represent activation/signaling.

The direct regulatory effects of tannins on the expression and secretion of core pro-inflammatory cytokines, as well as the targeted inhibitory trends toward key inflammatory mediators in the intestinal inflammatory microenvironment, are visually illustrated in Figure 3, which further clarifies the key targets of tannins in intervening in the pro-inflammatory cascade reaction of UC at the molecular level.

#### 3.1.2. Modulation of Pro-Inflammatory Signaling Cascades

Nuclear factor-kappa B (NF-*κ*B) constitutes a pivotal transcription factor family that regulates immune response and inflammation-associated gene expression through dimer complex formation, serving as a central mediator in inflammatory modulation [63]. During UC pathogenesis, aberrant NF-*κ*B activation facilitates transcriptional upregulation of pro-inflammatory cytokine genes, thereby propagating inflammatory cascades [64,65]. Evidence indicates that NF-*κ*B contributes to UC pathogenesis by upregulating expression of pro-inflammatory cytokines, including TNF-α and interleukin-1β (IL-1β) [66,67]. The regulatory effects of tannins on pro-inflammatory signaling pathways have been elucidated in various experimental settings. In an in vitro study using cultured macrophages, Xiaoyan Jia et al. [68] demonstrated that tannin treatment could modulate the PI3K/Akt and NF-*κ*B signaling pathways. This modulation effectively reduced the levels of pro-inflammatory mediators such as TNF-α and IL-1β at the transcriptional and translational levels, while concurrently elevating the expression of the anti-inflammatory cytokine interleukin-10 (IL-10). Although these findings are derived from a cellular model and cannot be directly extrapolated to the clinical setting, they provide important mechanistic insights into how tannins may exert their anti-inflammatory effects. Such in vitro evidence establishes a foundational rationale for further in vivo investigations and supports the potential development of tannin-based therapies for inflammatory conditions like UC.

**Table 1:** Effects of different tannin subclasses on inflammatory mediators.

Vegetal Species	Botanical Drugs	Inflammatory Factors	Mechanism of Action	Experimental Model	Literature
*Punica granatum* L. (Lythraceae)	Fructus	IL-6	By regulating the NF-*κ*B/STAT3 signaling pathway, the activation of proinflammatory factor IL-6-related inflammatory cascade reactions is inhibited.	Rat colon tissue (high-fat diet-induced); human keratinocytes	[66,69,70]
*Vitis vinifera* L. (Vitaceae)	Semen	COX-2, iNOS, TNF-α, IL-6	Acts on intestinal epithelial goblet cells, targeting the regulation of the FOXO1 signaling pathway, and intervening in the expression of factors such as COX-2 and iNOS, as well as inflammation responses mediated by TNF-α and IL-6.	Litopenaeus vannamei (in vitro cell model); porcine ruminal fermentation in vitro	[71,72,73]
*Fragaria* × *ananassa* (Weston) Duch. ex Rozier (Rosaceae)	Fructus	TNF-α	Activate NF-*κ*B, antagonize adiponectin	Murine 3T3-L1 adipocytes; in vitro bacterial culture (Listeria monocytogenes)	[74,75]
*Rosa multiflora* Thunb. (Rosaceae)	Radix	TNF-α, IL-6, IL-1β	Regulate inflammatory mediators, enzymes, and induce the expression of nitric oxide synthase and cyclooxygenase-2	NC/Nga mice (atopic dermatitis model)	[76,77]
*Castanea mollissima* Blume (Fagaceae)	Lignum	IL-1β, IL-6, TNF-α, IL-10	Regulate inflammatory mediators, induce the expression of NOS and COX-2, and enhance intestinal barrier function (tight junction proteins ZO-1, Claudin-1, and Occludin).	Human gut microbiota in vitro; pigskin gelatin cell model; rat serum in vitro	[49,78,79]
*Diospyros kaki* Thunb. (Ebenaceae)	Fructus	TNF-α, IL-6, IL-1β	Regulation of AKT protein expression in PI3K/AKT	Sprague-Dawley (SD) rats (high-cholesterol diet-induced); murine senescent model (D-galactose-induced)	[80,81,82]
*Crataegus pinnatifida* Bunge (Rosaceae)	Fructus	TNF-α, IL-6, IL-1β	Inhibiting excessive secretion of cellular inflammatory factors and intracellular ROS levels	Human respiratory epithelial cells; in vitro enzymatic reaction model	[83,84]
Chinensis galla (Anacardiaceae)	Galla	Epoxygenase	Inhibits cyclooxygenase activity and regulates cytokine expression.	Micropterus salmoides intestinal cells; broiler chicken peripheral blood cells; Apis mellifera intestinal epithelial cells	[85]
*Malus pumila* Mill. (Rosaceae)	Fructus	TNF-α, COX-2	Increase the expression levels of ZO-1 and occludin, and decrease the protein expression levels of NLRP3, apoptosis-associated speck-like protein (ASC), and effector protein caspase-1 in colon tissue, thereby inhibiting the activation of the NLRP3 inflammasome.	C57BL/6 mice (DSS-induced UC model); human gut microbiota in vitro	[86,87,88,89]
*Quercus variabilis* Blume (Fagaceae)	Fructus	TNF-α, IL-1β, IL-6	Reduce the secretion of pro-inflammatory factors	In vitro plant cell culture model (acorn nutlet callus)	[90]
*Arachis hypogaea* L. (Fabaceae)	Fructus	TNF-α, IL-6, IL-1β, TLR4, Myd88, NF-*κ*Bp65	Increase the expression of tight junction proteins (Claudin1, Occludin, and ZO-1) and intervene in the TLR4/Myd88/NF-*κ*B pathway.	C57BL/6 mice (DSS-induced UC model); murine intestinal epithelial cells	[91,92]
*Sorghum bicolor* (L.) Moench (Poaceae)	Herba	Free radical	Eliminate free radicals	In vitro chemical antioxidant model (DPPH/ABTS assay)	[93]
*Camellia sinensis* (L.) Kuntze (Poaceae)	Herba	TNF-α, IL-6, CRP	Regulation of arachidonic acid metabolism, hypoxia-inducible factor-1, platelet activation, etc.	RAW264.7 murine macrophages; human gingival epithelial/fibroblast 3D co-culture model	[94,95]
*Juglans regia* L. (Juglandaceae)	Fructus	IL-6, IL-12, TNF-α	Reduce oxidative stress and regulate inflammation-related signaling pathways	Human colon adenocarcinoma Caco-2 cells; in vitro bacterial culture	[96,97,98]
*Prunus armeniaca* L. (Rosaceae)	Sporoderm	TNF-α, IL-6, IL-1β	Binding to inflammation-related biomolecules	In vitro chemical antioxidant model; murine immune cells in vitro	[99,100]
*Rubus phoenicolasius* Maxim. (Rosaceae)	Folium	TNF-α, IL-6, IL-1β	Regulate the intestinal flora to promote the production of butyric acid	In vitro plant tissue culture (leaf extract); human gut microbiota in vitro	[74,101]

### 3.2. Counteracting Oxidative Stress: Dual Defense Mechanisms

UC exhibits strong pathophysiological associations with oxidative stress. UC patients demonstrate significant disruption of redox homeostasis [102,103,104,105], wherein the potent antioxidant properties of tannins may offer a potential therapeutic strategy for disease mitigation. Tannins exhibit robust antioxidant capabilities [24,106], primarily mediated through multiple mechanisms. It can enhance the activity of antioxidant enzymes such as superoxide dismutase (SOD) and GSH-Px, and reduce the levels of lipid peroxidation products such as malondialdehyde (MDA) [107], and alleviate oxidative damage.

The antioxidant effect of tannins has been verified in a variety of experiments. For example, Olatunde O.O. et al. [108] research showed that tannins extracted from *Cocos nucifera* L. (Arecaceae) shells can significantly reduce the conjugated diene, thiopropionic acid content, and aniseamine value in shrimp oil, effectively inhibiting the oxidation process of shrimp oil. Yan Tian et al. [109] further confirmed that tannins, as natural antioxidants, can scavenge DPPH free radicals and superoxide anions (O^2−^·), thereby improving antioxidant stress. The antioxidant mechanism of tannins also involves the regulation of the Nrf2/HO-1 signaling pathway in cells. By activating this pathway, tannins can upregulate the expression levels of downstream phase II detoxification enzymes and antioxidant enzyme genes, thereby enhancing the antioxidant stress resistance of cells [110,111].

**Figure 4:**
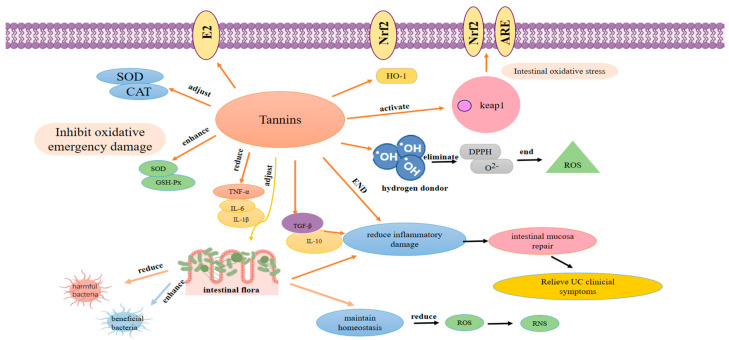
Schematic diagram of the antioxidant mechanisms of tannins in ameliorating UC-associated intestinal oxidative stress. This diagram illustrates the multifaceted antioxidant actions of tannins and their downstream protective effects on the intestinal tract in UC. Tannins exert antioxidant activity through two core pathways: (1) direct free radical scavenging: their phenolic hydroxyl groups act as hydrogen donors to eliminate ROS including ·OH, O_2_^−^·, DPPH· and interrupt free radical chain reactions; (2) potentiating endogenous antioxidant defense systems: tannins activate the Nrf2/HO-1 signaling pathway by dissociating Nrf2 from Keap1, thereby upregulating the expression and activity of key antioxidant enzymes (SOD, CAT, GSH-Px). By mitigating intestinal oxidative stress, tannins further inhibit the secretion of pro-inflammatory cytokines (TNF-α, IL-6, IL-1β), upregulate the anti-inflammatory cytokine IL-10, alleviate intestinal mucosal damage and promote mucosal repair. Additionally, tannins modulate the gut microbiota by reducing harmful bacteria and enriching beneficial taxa, which in turn maintains intestinal redox homeostasis and relieves clinical symptoms of UC, forming a synergistic antioxidant-anti-inflammatory-microbiota regulatory loop.

The dual antioxidant mechanisms of tannins in alleviating intestinal oxidative stress in UC—including direct free radical scavenging and the potentiation of endogenous antioxidant enzyme systems—as well as the downstream regulatory effects on oxidative stress-inflammation crosstalk and intestinal mucosal protection, are comprehensively visualized in Figure 4.

#### 3.2.1. Potentiation of Endogenous Antioxidant Enzymes

Tannins effectively control oxidative stress damage by enhancing the activity of SOD and glutathione peroxidase (GSH-Px), thereby exerting a therapeutic effect on UC [110]. Taking a UC mouse model as an example, after tannin treatment, the activity of these antioxidant enzymes in colon tissue significantly increased [112]. The mechanism of action may involve regulation of the transcription regulatory regions of related enzyme genes or modification of enzyme protein structures, thereby enhancing enzyme expression and activity. Hansuo Liu et al. [113] evaluated the effects of hydrolyzed tannin (HT) replacing zinc oxide (ZnO) on the growth performance and antioxidant status of weaned piglets. The results showed that, compared with the control group, the HT+ZnO group had significantly increased activities of catalase (CAT) and GSH-Px in serum, while MDA levels decreased, indicating a reduction in oxidative stress levels. Numerous studies have also confirmed this view [110,114,115].

#### 3.2.2. Direct Free Radical Scavenging Capacity

Tannins contain a large number of phenolic hydroxyl groups, which can act as hydrogen donors and bind with free radicals in the environment, thereby terminating the chain reaction triggered by free radicals and preventing further oxidation. This is consistent with previous research [116,117], and multiple studies have also confirmed this view, which identified the active site as the phenolic hydroxyl groups in tannin molecules. By providing hydrogen atoms to react with free radicals, tannins exert their antioxidant effects. Table 2 summarizes the antioxidant properties and mechanisms of different types of tannins

**Table 2:** Study on the antioxidant properties of different types of tannins.

Vegetal Species	Botanical Drugs	Free Radical Categories	Mechanism of Action	Literature
*Quercus acutissima* Carruth. (Fagaceae)	Fructus	ROS, DPPH·	Eliminate oxygen-free radicals in the body, increase antioxidant enzyme activity, and reduce oxidative stress levels in the body.	[44,118,119,120]
*Diospyros kaki*	Fructus	ROS	Releases active hydrogen atoms, reacts with free radicals, converts them into stable products, and interrupts free radical chain reactions.	[121,122]
*Vitis vinifera*	Semen	DPPH·	Eliminates DPPH free radicals, rescues cell survival after hydrogen peroxide treatment, reduces intracellular reactive oxygen species levels, and significantly reduces mRNA expression of pro-inflammatory cytokines after hydrogen peroxide treatment.	[72,73]
*Musa acuminata* Colla (Musaceae)	Fructus	ABTS·^+^	Elimination of ABTS^+^ free radicals	[123,124]
*Heritiera littoralis* Dryand. (Malvaceae)	Pericarpium	DPPH·, O_2_^−^·, ·OH	Eliminates DPPH free radicals, superoxide anion free radicals (O_2_^−^·), and hydroxyl free radicals (·OH)	[125]
*Acacia mearnsii* De Wild. (Fabaceae)	Cortex	ROS	Eliminate active oxygen, reduce singlet oxygen to less active triplet oxygen, reduce the possibility of oxygen free radical generation, chelate with metal ions, and reduce the catalytic effect of metal ions on oxidation reactions.	[126]
*Caragana korshinskii* Kom. (Fabaceae)	Herba	ABTS·^+^, O_2_^−^·, DPPH·	Activate the Nrf2/ARE signaling pathway, promote Nrf2 nuclear translocation and activation, and initiate the expression of antioxidant stress-related proteins and enzymes.	[127]
*Nelumbo nucifera* Gaertn. (*Nelumbonaceae*)	Pericarpium	DPPH·, O_2_^−^·, ·OH	Activate the Nrf2/ARE signaling pathway, promote Nrf2 dissociation, and increase the transcriptional level of the antioxidant protection gene HO-1.	[128]
*Castanea mollissima*	Lignum	O_2_^−^·, ·OH	Eliminate free radicals, reduce the concentration of MDA in urine, reduce DNA damage in serum lymphocytes, and increase SOD and GSH-Px in plasma.	[79,129]
*Dysoxylum excelsum* Blume (Meliaceae)	Lignum	ABTS·^+^, DPPH·, O_2_^−^·	Reduces MDA content, increases T-SOD and GSH-Px activity, and improves the body’s antioxidant capacity.	[130]
*Cercis chinensis* Bunge (Fabaceae)	Folium	DPPH·	Elimination of DPPH free radicals	[131]
*Citrus reticulata* Blanco (Rutaceae)	Pericarpium	OH·, DPPH·	Elimination of hydroxyl radicals and DPPH free radicals	[132]
*Plotytarya strohilacea* Sieb et Zuce (Juglandaceae)	Fructus	ABTS·^+^, ·OH, O_2_^−^·	Significantly increases CAT activity and T-AOC, removes free radicals from the body, and reduces plasma CREA and UN levels	[133]
*Prunus armeniaca*	Pericarpium	DPPH·, O_2_^−^·, ·OH	Regulate the Keapl-Nrf2/ARE signaling pathway to increase the expression of antioxidant proteins in the body.	[99,134]
*Castanea mollissima*	Bractea	DPPH·, ABTS·^+^	Eliminates DPPH free radicals and ABTS^+^ free radicals and has a certain Fe^3+^ reduction capacity.	[135]
*Argentina anserina* (L.) Rydb. (Rosaceae)	Herba	DPPH·	Elimination of DPPH free radicals	[136,137]
*Juglans regia*	Endopleura	ROS	Release H+ competes with free radicals to prevent chain reactions.	[138,139]
*Punica granatum*	Pericarpium	DPPH·, ·OH, O_2_^−^·	Increase the concentration of SOD and CAT in serum while reducing MDA concentration.	[140,141,142,143]
Chinensis galla	Galla	DPPH·, O_2_^−^·, ·OH	It has excellent antioxidant properties, reducing oxidized vitamin C (Vc) to its reduced form, thereby regenerating Vc. It upregulates the expression of antioxidant enzyme genes such as SOD, CAT, and GSH-Px, increases enzyme synthesis, and enhances the body’s antioxidant capacity.	[85,144,145,146]
*Morella esculenta* (Buch.-Ham. ex D. Don) I. M. Turner (Myricaceae)	Fructus	ABTS·^+^, DPPH·, ·OH	Eliminates ABTS^+^ radicals, DPPH radicals, hydroxyl free radicals, and has a certain Fe3+ reduction capacity.	[147]
*Rhodomyrtus tomentosa* (Aiton) Hassk. (Myrtaceae)	Fructus	O_2_^−^·, ·OH	Within a certain concentration range, the antioxidant activity of myrtle fruit tannins shows a good dose–response relationship with their concentration.	[148,149]
*Sorghum bicolor*	Semen	DPPH·, ABTS·^+^	Eliminates DPPH free radicals and ABTS^+^ free radicals, and has a certain Fe^3+^ reduction capacity.	[150,151,152,153,154]
*Ficus altissima* Blume (Moraceae)	Fructus	DPPH·, ABTS·^+^	Eliminates ROS, DPPH free radicals, and ABTS^+^ free radicals	[155]
*Corymbia citriodora* (Hook.) K.D.Hill&L.A.S.Johnson (Myrtaceae)	Cortex	·OH	Eliminate hydroxyl radicals (·OH)	[156,157]
*Quercus × leana* Nutt. (Fagaceae)	Fructus	DPPH·, ·OH	Eliminates DPPH free radicals and hydroxyl free radicals	[120]
*Kalopanax septemlobus* (Thunb.) Koidz. (Araliaceae)	Folium	ABTS·^+^	Eliminates ABTS^+^ radicals and DPPH radicals, with higher elimination activity for ABTS^+^ radicals than for DPPH radicals	[158]
*Dalea purpurea* Vent. (Fabaceae)	Herba	ROS	Affects certain enzymes related to redox reactions	[159,160]
*Taraxacum mongolicum* Hand.-Mazz. (Asteraceae)	Herba	O_2_^−^·, ·OH	Activate SOD, GSH-Px, and CAT	[161,162]

### 3.3. Gut Microbiota Remodeling: Pathobiont Suppression and Symbiont Enrichment

The balance of the gut microbiota is crucial for maintaining overall health, particularly in the context of the pathogenesis and treatment of UC. Tannins, as a class of natural bioactive metabolites, exhibit unique efficacy in regulating the gut microbiota, primarily through two key mechanisms: inhibiting the growth of harmful bacteria and promoting the growth of beneficial bacteria [163,164,165,166]. To further explore the role of tannins in maintaining gut microbiota balance in UC, this study will provide a detailed analysis from these two perspectives.

**Figure 5:**
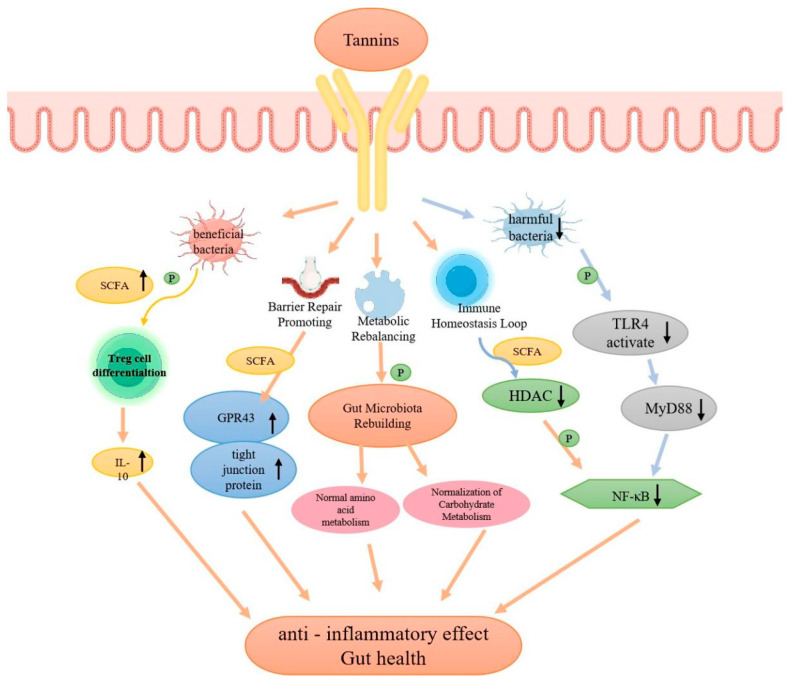
Schematic diagram of the mechanism by which tannins regulate the intestinal microbiota. This diagram illustrates the multi-layered regulatory effects of tannins on gut microbiota and the subsequent formation of a gut health homeostasis loop in UC, with key clarifications on tannin binding and immune interactions: tannins do not directly interact with antibodies; their biological effects are initiated by targeting and remodeling the gut microbiota (the core upstream mechanism), and they exert indirect cellular regulation by binding to GPR43 (G protein-coupled receptor 43) on intestinal epithelial cells and immune cells (the only well-characterized receptor for tannin downstream signaling). Tannins first inhibit the colonization and proliferation of intestinal pathobionts (e.g., *Escherichia coli*) and enrich beneficial symbionts (e.g., *Lactobacillus*, *Bifidobacterium*), which promotes the production of short-chain fatty acids (SCFAs) by beneficial bacteria. SCFAs then activate GPR43 on intestinal cells, further inhibiting the TLR4/MyD88/NF-*κ*B inflammatory signaling pathway, reducing pro-inflammatory cytokine secretion and promoting the differentiation of Treg cells to maintain immune homeostasis. Concurrently, tannin-induced microbiota remodeling upregulates the expression of intestinal tight junction proteins (e.g., ZO-1, Occludin) to repair the intestinal barrier, normalizes intestinal amino acid and carbohydrate metabolism, and ultimately forms a synergistic loop of microbiota remodeling-barrier repair-immune/metabolic homeostasis to alleviate intestinal inflammation and improve UC symptoms. Arrow explanation: Downward arrows (↓) indicate downregulation or inhibition; upward arrows (↑) indicate upregulation or activation; solid arrows (→) represent activation/signaling.

The mechanistic pathways underlying tannin-mediated gut microbiota remodeling in UC—including pathobiont suppression, symbiont enrichment and subsequent intestinal barrier repair and immune homeostasis regulation—are systematically visualized in Figure 5; it is critical to clarify that tannins exert these effects primarily by binding to G protein-coupled receptors (GPR43) on intestinal epithelial and immune cells (the key receptor for short-chain fatty acid signaling) and do not directly interact with antibodies or trigger antibody-mediated immune responses, with their immunomodulatory effects being indirect and microbiota-dependent.

#### 3.3.1. Inhibition of Enteropathogenic Colonization

UC patients exhibit increased levels of harmful gut bacteria such as *Escherichia coli* and other Enterobacteriaceae. Tannin metabolites can inhibit the growth of harmful bacteria, thereby exerting a therapeutic effect on UC [167,168]. Its mechanism of action may be related to the destruction of cell membranes and the inhibition of key enzyme activity. Researchers had also found that tannins have antibacterial effects on *Escherichia coli*, with their action sites being the bacterial cell membrane, cell wall, and key metabolic enzymes within the cell [169,170,171]. Additionally, tannins can inhibit pathogenic bacteria in water and have a resistant effect against pathogenic microorganisms that infect crustaceans. Qinglian Qiu et al. [172] found that Galla chinensis extract (rich in tannins) exhibited the best antibacterial effect against *Vibrio parahaemolyticus*, through in vitro experiments comparing extracts from Chinensis galla, Mume fructus, Asparagi radix, and Fraxini radix, its antibacterial activity was significantly superior to that of other tested drugs.

#### 3.3.2. Promotion of Beneficial Taxa Proliferation

In the pathological state of UC, the number of beneficial bacteria, such as *Bifidobacterium* and *Lactobacillus,* in the intestine is significantly reduced. Tannins promote the growth and reproduction of these beneficial bacteria and can exert positive effects by improving the intestinal microecology. For example, in animal experiments, mice treated with tannins showed a significant increase in the number of *Bifidobacterium* in their intestines [59,173]. It is currently speculated that tannins exert their effects in the following two ways: on the one hand, they can supply nutrients to beneficial bacteria, helping them to grow and reproduce better; on the other hand, tannins can optimize the microenvironment of the intestine, making it more suitable for the survival of beneficial bacteria [174,175].

Not only that, tannins have a wide range of effects. They are not only beneficial to the intestines of animals or mammals suffering from UC, but also play a positive role in fish. Specifically, tannins can increase the number of beneficial bacteria in the intestines of fish while inhibiting the growth of harmful bacteria [176,177], thereby effectively regulating the microecological balance of the fish’s intestines and further promoting the intestinal health of the animals. As demonstrated by Kai Peng et al. [178], when 400 mg/kg of condensed tannins were added to the feed, the gut microbiota structure of sea bass underwent significant changes. Specifically, condensed tannins increased the relative abundance of *Clostridium* and *Spirochaeta* in the bacterial community while reducing the relative abundance of *Aeromonas*, thereby decreasing the secretion of intestinal endotoxins, improving intestinal permeability in sea bass, and ultimately enhancing their intestinal health.

The regulatory effects of tannins from diverse vegetal species on intestinal beneficial bacteria, as well as the corresponding experimental models, administration routes, tannin application forms and underlying action mechanisms, are systematically summarized in Table 3. These findings, validated by in vitro fecal microbiota fermentation, in vivo animal models (including classic UC models and diet-induced metabolic disorder models) and human clinical samples, confirm that tannins (used as plant extracts via oral administration) can reshape gut microbiota homeostasis by enriching key beneficial bacteria (e.g., *Lactobacillus*, *Bifidobacterium*), which further provides experimental evidence for the microbiota-mediated anti-inflammatory effect of tannins in UC.

**Table 3:** Effects of tannins from different sources on the gut microbiome and their mechanisms of action.

Vegetal Species	Types of Beneficial Bacteria	Mechanism of Action	Experimental Model	Administration Route	Tannin Form	Literature
*Quercus acutissima*	*Lactobacillus*, *Bifidobacterium*	Reduce oxidative stress, promote the reproduction of beneficial bacteria, and inhibit the growth of harmful bacteria.	Broiler chickens (in vivo); in vitro antioxidant model	Oral (dietary supplementation)	Plant extract (valonia tannin extract)	[118]
*Diospyros kaki*	*Lactobacillus*, *Bifidobacterium*	Significantly promotes the growth of beneficial bacteria such as *Lactobacillus* and *Bifidobacterium*.	Sprague-Dawley (SD) rats (normal/high-cholesterol diet)	Oral (gavage)	Plant extract (persimmon tannin extract)	[82,179]
*Mangifera indica* L. (Anacardiaceae)	*Lactobacillus*	Beneficial regulation of bacteria associated with the metabolism of bioactive gallic acid tannin metabolites	Human fecal microbiota (in vitro fermentation); lean/obese human volunteers	Oral (dietary supplementation)	Plant extract (mango polyphenol/tannin extract)	[180,181]
*Rubus idaeus* L. (Rosaceae)	*Bifidobacterium*, *Blautia*, *Ruminococcus*	Alter the composition of the gut microbiota to promote the growth of beneficial bacteria in the intestines.	Wistar rats (in vivo)	Oral (dietary supplementation)	Plant extract (raspberry pomace tannin extract)	[182,183]
*Vitis vinifera*	*Bifidobacterium*, *Akkermansia muciniphila*	It can increase beneficial bacteria (such as *Bifidobacterium* and *Akkermansia muciniphila*) and reduce harmful bacteria.	Weaned piglets (in vivo); C57BL/6 mice (D-galactose-induced aging model)	Oral (dietary supplementation/gavage)	Plant extract (grape seed tannin extract)	[184,185,186]
*Camellia sinensis*	*Akkermansia muciniphila*, *Alloprevotella, Bacteroides*, *Faecalibaculum*	Reduce the abundance of harmful bacteria and increase the abundance of beneficial bacteria.	Human fecal microbiota (in vitro); mouse colitis model	Oral (gavage/tea infusion)	Plant extract (tea polyphenol/tannin extract)	[187,188,189]
*Punica granatum*	*Prevotellaceae*, *Lactobacillus*	Enhance gut microbiota diversity and increase the relative abundance of beneficial bacteria.	SD rats (high-fat diet-induced colonic damage); human fecal microbiota (in vitro)	Oral (gavage/dietary supplementation)	Plant extract (pomegranate peel tannin extract)	[70,190,191]
*Malus pumila*	*Bifidobacterium*, *Lactobacillus*,	Downregulates the pro-inflammatory factor TNF-α, upregulates the inflammatory and immunosuppressive factor IL-10, and increases the expression levels of ZO-1 and occludin in colon tissue.	C57BL/6 mice (DSS-induced UC model); human gut microbiota (in vitro)	Oral (gavage)	Plant extract (apple polyphenol/tannin extract)	[88,192,193,194,195,196]
*Rubus fruticosus* L. (Rosaceae)	*Agathobacter rectalis*, *Bacteroides fragilis*	By reducing the expression levels of inflammatory cytokines such as IL-1, IL-6, and COX-2, it promotes the growth of beneficial bacteria in the gut.	C57BL/6 mice (high-fat diet model); in vitro bacterial culture	Oral (gavage)	Plant extract (blackberry tannin/anthocyanin extract)	[197,198,199]
*Castanea mollissima*	*Lactobacillus*, *Bifidobacterium*, *Faecalibacterium*	Has a significant inhibitory effect on *Clostridium perfringens*.	Broiler chickens (in vivo); zebrafish (intestinal inflammation model); in vitro rumen fermentation	Oral (dietary supplementation)	Plant extract (chestnut tannin extract)	[79,200,201,202]
*Juglans regia*	*Lactobacillus aviarius*, *Lactobacillus agilis*	Enhance gut microbial diversity and increase the abundance of beneficial bacteria.	Human volunteers (in vivo); human colon adenocarcinoma Caco-2 cells (in vitro)	Oral (dietary supplementation)	Plant extract (walnut pellicle tannin extract)	[203,204]
*Vaccinium uliginosum* L. (Ericaceae)	*Lactobacillus*, *Bifidobacterium*	Influences the ability of gut microbiota to metabolize carbohydrates, amino acids, and energy, thereby regulating the abundance and diversity of gut microbiota.	C57BL/6 mice (high-fat/high-sucrose diet model)	Oral (gavage)	Plant extract (blueberry proanthocyanidin/tannin extract)	[205,206]
*Ziziphus jujuba* var. *Inermis* (Bunge) Rehder (Rhamnaceae)	*Lactobacillus*, *Bifidobacterium*	To enhance the diversity of the gut microbiota in UC mice, increase the abundance of beneficial bacteria, and thereby regulate the gut microbiota in UC mice.	C57BL/6 mice (UC model); honey bees (in vivo)	Oral (gavage/dietary supplementation)	Plant extract (jujube powder/polyphenol extract)	[207,208,209]
*Fragaria* × *ananassa*	*Agathobacter*, *Blautia*, *Bifidobacterium*	By promoting the proliferation of beneficial bacteria, inhibiting the overgrowth of harmful bacteria, and optimizing the structure of the intestinal microbiota	Wistar rats (high-fructose diet model); in vitro Listeria monocytogenes culture	Oral (dietary supplementation)	Plant extract (strawberry tannin/ellagitannin extract)	[210]
*Pinus yunnanensis* Franch. (Pinaceae)	*Lactobacillus*, *Bifidobacterium*	Effectively improves the morphology, diversity, and structural composition of the intestinal microbiota in piglets, alleviates intestinal mucosal damage, and restores intestinal barrier function.	Weaned piglets (in vivo); human fecal microbiota (in vitro fermentation)	Oral (dietary supplementation)	Plant extract (pine bark tannin extract)	[211,212]
Chinensis galla	*Lactobacillus*, *Bifidobacterium*	Improve intestinal tissue structure, optimize intestinal flora, and protect intestinal health.	Broiler chickens (aflatoxin B1-challenged model); Micropterus salmoides (in vivo); Apis mellifera (in vivo)	Oral (dietary supplementation/gavage)	Plant extract (galla chinensis tannic acid extract)	[85,144,145,213]

## 4. Discussion

### 4.1. Challenges in the Treatment of IBD

IBD presents multiple challenges in clinical management. First, the complex etiology of IBD makes it difficult to target treatment at a single factor. Secondly, while mucosal healing reduces recurrence, it does not cure the disease [214]. Deep remission and histological healing have emerged as new targets, though their long-term efficacy and drug feasibility remain to be validated. Furthermore, biological agents have limitations, such as systemic side effects(e.g., increased risk of opportunistic infections, infusion reactions, and hepatotoxicity) and the inability to induce localized mucosal responses [215]. While genetically modified microbial therapies are under investigation, they pose biosafety risks, they pose biosafety risks, including the potential for horizontal gene transfer or unintended environmental release.

Additionally, IBD patients experience increased nutritional demands coupled with reduced intake, making nutritional therapy indispensable yet requiring individualized adjustments. Finally, the heterogeneity and recurrent nature of IBD complicate the establishment of uniform treatment endpoints, necessitating large-scale studies to optimize therapeutic approaches. These challenges underscore the complexity and long-term nature of IBD management.

Natural compounds demonstrate unique advantages in IBD treatment. Their multi-targeted mechanisms simultaneously regulate immunity, improve gut microbiota, and repair the mucosal barrier; with relatively fewer side effects, they are suitable for long-term use [216,217,218]. Certain components, such as berberine [219,220] and curcumin [221] possess anti-inflammatory and antioxidant properties that effectively alleviate symptoms. Integrating traditional Chinese medicine with Western medicine can reduce the dosage of Western drugs and lower treatment costs. Natural compounds offer new therapeutic options for IBD, particularly for patients unresponsive to conventional treatments.

### 4.2. Dual Mechanisms of Tannins: Synergistic Protection of the Intestinal Barrier Through Antioxidant and Anti-Inflammatory Effects and Regulation of Immune Balance

Tannins, as naturally occurring active components, offer a new perspective for UC treatment through “multi-pathway regulation and multi-target intervention”. The role of tannins in improving UC is based on the synergistic action of dual antioxidant and anti-inflammatory mechanisms. In terms of antioxidant activity, tannins directly scavenge hydroxyl radicals, superoxide anions, and other ROS through their phenolic hydroxyl structures, thereby interrupting the oxidative stress cascade reaction [15]. On the other hand, it enhances the body’s endogenous antioxidant system by upregulating the expression of antioxidant enzyme genes such as SOD and GSH-Px, thereby reducing oxidative stress-induced damage to intestinal epithelial cells and maintaining the integrity of the intestinal barrier. At the cellular level, tannins can inhibit oxidative stress-induced apoptosis of intestinal epithelial cells, promote the expression of tight junction proteins (such as ZO-1 and Occludin), and delay the destruction of the intestinal mucosal barrier in UC [115]. In terms of anti-inflammatory effects, tannins can inhibit the expression of key inflammatory cytokines, including pro-inflammatory factors. They can inhibit the activation of signaling pathways such as NF-*κ*B and MAPK [46], thereby reducing the transcription and secretion of pro-inflammatory cytokines such as TNF-α, IL-1β, and IL-6; simultaneously. They regulate immune cell function, suppress excessive activation of macrophages, promote the immune suppressive function of Treg cells, and restore intestinal immune balance, thereby alleviating inflammatory responses at their source [222]. Additionally, their precise regulation of immune cell function provides a robust theoretical foundation for their application in the treatment of UC.

### 4.3. Clinical Potential and Limitations of Tannins

The bioactivity of tannins (e.g., antioxidant, anti-inflammatory, and gut microbiota-regulating effects) is closely related to their molecular structures, such as the number of phenolic hydroxyl groups, degree of polymerization, and structural differences between hydrolyzable and condensed tannins, which determine their binding affinity to biological targets and functional specificity.

Although tannins possess polyphenolic antioxidant and immunomodulatory potential in anti-inflammatory therapy, their clinical translation faces significant obstacles. The primary challenges include low oral bioavailability due to large molecular weight, poor water solubility, and rapid intestinal metabolism, resulting in insufficient blood concentrations. Additionally, tannins readily form insoluble complexes with gastrointestinal proteins and metal ions (such as iron and calcium), not only diminishing therapeutic efficacy but also posing risks of iron absorption impairment and osteoporosis.

Although clinical studies on tannin for UC treatment remain limited, some trials have demonstrated symptom relief in IBD, with improvements in inflammatory markers [223,224]. For instance, in one study where IBD animal models were administered tannin, both in vivo and in vitro results demonstrated a dose-dependent dual effect of tannin on DSS-induced IBD. At protective doses, gallic acid enhanced colonic mucus secretion, inhibited bacterial penetration of the colonic epithelium, and reduced expression of inflammatory factors in intestinal tissues [225]. These positive changes indicate that tannins have potential application value in the clinical treatment of UC, making them worthy of further in-depth research.

In the future, more advanced technological methods, such as single-cell sequencing and proteomics, will be needed to thoroughly investigate the specific targets and mechanisms of action of tannins in the body. Clarifying the precise interaction between tannins and various intracellular signaling pathways will facilitate the development of more precise tannin-based treatment strategies, thereby improving the specificity and efficacy of treatment. In the clinical application of tannins, efforts can focus on enhancing their oral bioavailability through nanodelivery systems, while modifying their structure to improve permeability and stability. Further exploration of the synergistic effects of tannins with other novel drugs, botanical drugs, or therapeutic modalities is also warranted. By screening for optimal combination regimens and dose ratios, more efficient and less toxic combination therapy protocols can be developed to address the personalized treatment needs of different UC patients, thereby improving treatment outcomes and enhancing their quality of life.

## 5. Conclusions

In conclusion, this review systematically consolidates the current understanding of the therapeutic potential of tannins in UC. The evidence strongly supports that tannins exert their beneficial effects through a synergistic dual-action mechanism. They act as potent antioxidants by directly scavenging ROS and upregulating endogenous antioxidant enzymes, while simultaneously functioning as anti-inflammatory agents by suppressing key signaling pathways (e.g., NF-*κ*B, MAPK) and downregulating pro-inflammatory cytokines (e.g., TNF-α, IL-1β, IL-6). This integrated regulation of the gut’s inflammatory-oxidative homeostasis is further complemented by their ability to remodel the gut microbiota, suppressing pathogenic bacteria and promoting the growth of beneficial taxa, thereby reinforcing intestinal barrier integrity.

Despite these promising multi-targeted effects, the clinical translation of tannins faces significant hurdles that must be acknowledged as limitations of the current research landscape. A primary challenge is the limited oral bioavailability of native tannins due to their high molecular weight, poor aqueous solubility, and extensive metabolism within the gastrointestinal tract. Consequently, most of the compelling evidence for their efficacy is derived from in vitro and in vivo animal models, with a notable scarcity of well-designed, large-scale human clinical trials to confirm these effects in patients with UC. Furthermore, the field is complicated by the inherent structural heterogeneity of tannins derived from different plant sources, making it difficult to standardize extracts and compare results across studies.

Therefore, future research should prioritize addressing these limitations to pave the way for clinical applications. Key directions include: the development of advanced drug delivery systems, such as nanoparticles or liposomes, to enhance the oral bioavailability and targeted delivery of tannins to the colon; the design and execution of rigorous, randomized controlled trials to evaluate the efficacy and safety of well-characterized tannin formulations in UC patients. By overcoming these challenges, tannin-based therapeutics hold promise as a novel class of natural agents for the precision management of UC.

## Data Availability

The original contributions presented in this study are included in the article. Further inquiries can be directed to the corresponding author.

## Abbreviations

The following abbreviations are used in this manuscript:

·OH	Hydroxyl radical
ABTS·^+^	2,2′-Azino-bis(3-ethylbenzothiazoline-6-sulfonic acid) radical cation
Akt	Protein kinase B
AMPK	Adenosine monophosphate-activated protein kinase
ARE	Antioxidant response element
ASC	Apoptosis-associated speck-like protein containing a CARD
CAT	Catalase
COX-2	Cyclooxygenase-2
CREA	Creatinine
DPPH·	1,1-Diphenyl-2-picrylhydrazyl radical
DSS	Dextran sulfate sodium
FOXO1	forkhead box O1
GR	Glutathione reductase
GSH-Px	Glutathione peroxidase
HO-1	Heme oxygenase 1
HT	Hydrolyzed tannin
IF	Immunofluorescence
IHC	Immunohistochemistry
IL	Interleukin
IL-10/6/1*β*	Interleukin-10/6/1*β*
iNOS	Inducible nitric oxide synthase
LPS	Lipopolysaccharide
MDA	Malondialdehyde
Myd88	Myeloid differentiation factor 88
NF-*κ*B	Nuclear factor kappa-B
NLRP3	Nod-like receptor protein 3
Nrf2	Nuclear factor erythroid 2-related factor 2
O_2_^−^·	Superoxide
PI3K	Phosphatidylinositol 3-kinase
ROS	Reactive oxygen species
SOD	Superoxide dismutase
STAT3	Signal transducer and activator of transcription 3
T-AOC	Total antioxidant capacity
TLR4	Toll-like receptor 4
TNBS	2,4,6-Trinitrobenzene sulfonic acid
TNF-*α*	Tumor necrosis factor-*α*
UC	Ulcerative colitis
UN	Urea nitrogen
ZnO	Zinc oxide
ZO-1	Zonula occludens-1
